# Psychometric properties of the complete version of the World Health Organization Quality of Life Assessment (WHOQOL-OLD): reduced response scale

**DOI:** 10.1186/s41155-018-0084-1

**Published:** 2018-01-30

**Authors:** Rômulo Lustosa Pimenteira de Melo, Edivan Gonçalves da Silva Júnior, Rafaella Queiroga Souto, Ísis Simões Leão, Maria do Carmo Eulálio

**Affiliations:** 1 Cidade Universitária (UFPB), s/n - Castelo Branco III, João Pessoa-PB, 58051-900 Brazil; 2 (UEPB), R. Domitila Cabral de Castro, 38 - Universitário, Campina Grande-PB, 58429-570 Brazil; 3 (UFPE), Av. Prof. Moraes Rego, 1235 - Cidade Universitária, Recife-PE, 50670-901 Brazil; 4 R. Aprígio Veloso, 882 - Universitário, Campina Grande-PB, 58429-900 Brazil

**Keywords:** Health of the elderly, Quality of life, Validation studies

## Abstract

This study sought to compare evidence of the factorial validity of the adapted version of the World Health Organization Quality of Life (WHOQOL-OLD) scale (three response options) with the original version of the scale. We included two populations of individuals age 60 years or older from northeast Brazil. The majority of participants were women who had an elementary-level education. The first population comprised 335 elderly persons who completed the original version of the scale, and the second was composed by 265 elderly persons who completed the shortened scale. Results indicated better adjusting on the reduced scale and showed items with lower error probability in assessment of elderly persons with lower quality of life.

## Background

The term quality of life (QL) entails a complex, multidimensional, and multidetermined construct that is usually compared with well-being and happiness, but QL can present a variety of definitions and forms to be measured (Gordia, Quadros, Oliveira, & Campos, 2011). One of the most-used definitions was created by the World Health Organization (WHO), which defines QL as “individuals’ perception of their position in life in the context of the culture and value systems in which they live and in relation to their goals, expectations, standards and concerns” (WHOQOL Group, 1995).

The rise in the life expectancy of the world population and the need to provide good life conditions for older individuals pose a challenge to the scientific community to study quality of life in older people and identify what factors may influence their life (Leão, 2012; Liu et al., 2013; Lucas-Carrasco, Laidlaw, & Power, 2011). Quality of life measurement has been gaining attention, and although such measures are widely used, most instruments are not easy to understand and validate for older populations, which presents specific challenges and higher prevalence of cognitive and health problems and low education (Chachamovich, Fleck, Clarissa, & Power, 2008). For this reason, improvement of instruments with which we can measure elderly persons’ QL in terms of validity and in a consistent, easily understandable form is needed (Caballero et al., 2013).

To evaluate QL and its related difficulties, the WHO created the WHOQOL-100 and, subsequently, the WHOQOL-BREF, which are used around the world. However, these instruments do not include elements specific to older populations. Therefore, a study using the WHO instruments was carried out to investigate QL among Brazilian old people. Results indicated that in the studied group, QL is especially related to well-being, positive thoughts, and health (Fleck, Chachamovich, & Trentini, 2003).

Results showed features of the older population that should be included in instruments for appropriate measurement of QL. To address these features, the WHOQOL group created a specific questionnaire for elderly persons, known as WHOQOL-OLD. This questionnaire follows the same method of other instruments of QL created by the WHO (Fleck, Chachamovich, & Trentini, 2006).

The WHOQOL instrument has been used in different countries and has presented good psychometric performance. A study carried out among 286 elderly persons concluded that both WHOQOL-OLD and WHOQOL-BREF have good internal consistency and construct validity (Lucas-Carrasco et al., 2011). The Chinese version of WHOQOL-OLD also had good internal construct and discriminating validity (Liu et al., 2013). Conrad, Matschinger, Riedel-Heller, von Gottberg, and Kilian (2014) conducted a validation study among a representative sample of German old people and also concluded that the instrument had adequate psychometric proprieties. The result of the factorial analysis of a study conducted with older adults living in Finland and Poland showed the exclusion of some items of the instrument and its division into two factors, the first factor with autonomy and sensory functioning; and the second factor with satisfaction in past, present and future activities, death, and dying. Both factors showed good internal, convergent, and discriminant validity (Caballero et al., 2013).

In Brazil, the validation study of WHOQOL-OLD was developed with a sample of 424 old people from Porto Alegre–RS, and analyses enabled the researchers to consider the scale as having good psychometric general performance with satisfactory internal consistency, discriminant validity, concurrent validity, and reliability in test-retest validity but with fragility in the facets “sensory abilities” (SA) and intimacy (Fleck et al., 2006). Posteriorly, we performed analysis with Rash models in the same population and few adequacies were confirmed for items’ curves for SA and “death and dying.” To solve the problem, categories of responses for items 4, 5, 9, and 20 were reduced from five to four options, and items 3 and 18 were excluded. After exclusion, results presented better indicators (Chachamovich et al., 2008).

Because previous studies had varied results and used specific samples from particular regions, studies performed among old people from northeast Brazil sought to evaluate psychometric properties of the WHOQOL-OLD scale (Leão, 2012). One study suggested that scale could have better psychometric performance if the five answer categories of the instrument were condensed into three: the first would be the union of responses 01 and 2, the second the response category would be response 3, and the third would be responses 4 and 5. Although the extracted model with a factorial exploratory analysis of the three-response option would be a better adjustment when compared with the five-response model, the criterion of root-mean-square error of approximation (RMSEA) was still not ideal and suggested that new improvements should be tested.

The efficacy of reducing the WHOQOL instrument from five to three categories was also proved in a low-education population (Chachamovich et al., 2008) and one with intellectual deficiencies (Fang et al., 2011). These findings suggest that this instrument could be successful in populations that may have difficulty understanding the scale with five response points. This can occur with old people because those with a low educational level tend to have greater difficulty discriminating their responses when presented with a high number of options; that, in turn, could compromise response validity and consistency (Chachamovich et al., 2008). These findings indicate that having five response categories available can expose individuals to a prejudicial effort given that the scale tends to present better psychometric performance with three response categories. These results were indicated through Confirmatory Factor Analysis and Item Response Theory, model of Samejima (Fang et al., 2011). Based on these considerations, it is possible that the higher the number of response options, the more difficulty old people will have in choosing the category that clearly expresses their option. Therefore, WHOQOL-OLD applied to older people in northeast Brazil or other less educated populations should use a Likert-type three-point scale.

We highlight, however, that an important limitation in the studies by Chachamovich et al. (2008) and Leão (2012), which verified better adjustment to three response options, is that the scale was applied in its original version (five response options) and then reduced only during psychometric analysis. This procedure does not prevent older people from finding it difficult to answer items because reduction was made posteriorly. In this sense, further studies are needed to apply the reduced response scale to old people in order to compare the two versions and analyze behavior of responses in terms of QL in old people.

This need motivated us to perform our study. Our objective was to compare evidence of validity of the three-response WHOQOL-OLD scale with the original, five-response version and to verify behavior of items from the adapted version by the use of item response theory.

## Methods

The study was developed with two different samples of elderly residents in the countryside of Paraiba, Brazil. First, the WHOQOL-OLD scale was applied in its original version with five response options, and in the second, an adapted version of the scale, following the suggestion by Leão (2012), with three responses was administered. For both samples, we used a non-probabilistic approach with accessibility and convenience criteria. The first application included 355 old people; 66.9% were women, 46.9% were married or lived with a partner, the mean age was 73.9 years (SD, 7.34); and 84.2% had an elementary education. The second application included 265 elderly persons: 68.7% women, 51.0% married or living with a partner, mean age of 75 years (SD, 6.14), and 85.5% with an elementary education.

To select participants, we adopted the following criteria: minimal age of 60 years and agreement to participate in the study. We excluded old people who had suggestive cognitive deficit according to the Mini-Mental Status Examination (MMSE), and severe hearing deficit (observed by self-report and/or the own researcher).

### Instruments

To characterize participants, we applied a sociodemographic questionnaire with items that verified age, sex, formal education, and marital status.

The MMSE was used for tracking participants’ cognition. This instrument is commonly used in research among this population and can be easily and rapidly applied. The exam includes 30 questions (maximal score of 30) (Folstein, Folstein, & McHugh, 1975). We used the adapted version for the Brazilian population, evaluated according to formal education criteria (Brucki, Nitrini, Caramelli, Bertolucci, & Okamoto, 2003).

The WHOQOL-OLD scale evaluates QL in old people. The instrument was translated and validated for the Brazilian population (Chachamovich et al., 2008; Fleck et al., 2006). It has 24 items with responses formulated on a Likert-type scale: “nothing” (1), “rarely” (2), “medium” (3), “very” (4), and “completely” (5). Six domains are represented: sensory function (SF); intimacy (Int); autonomy (Aut); social participation (SP); past, present, and future activities (PPF); and death and diet (D&D). In the reduced version (second sample), we adopted the suggestion by Leão (2012) to use three response options: “nothing” (0), “medium” (1), and “completely” (02).

### Procedures

In the two samples, data were collected with the collaboration of undergraduate students appropriately trained to apply instruments for the study. The data collection was done at the homes of the elderly participants. Initially, participants were informed of the study objective and the confidentiality of their identification and data provided; they state their willingness to participate in the study and signed a consent form and agreed to posterior instrument application.

The studies followed procedures observed by the 466/2012 resolution of national health council and were approved by the Ethical and Research Committee of the Universidade Estadual da Paraíba, number 0505.0.133.000–10 (study that applied original scale version) and number 20599513.9.0000.5187 (study that applied reduced version). The WHOQOL-OLD instrument is freely available for research use. The modified version proposed in this article will also be available with no copyrights.

### Data analysis

The dimensionality hypothesis was verified by the confirmatory factorial analysis (CFA) with the Lavaan package (Rosseel, 2012) in software R. Considering the following adjustment index: *χ*^2^/df index, Tucker-Lewis index (TLI), comparative adjustment index, and approximate mean square error (RMSEA). For this, Marôco (2014) suggests the following criteria: *χ*^2^/df between 2 and 5 poor adjustment and between 1 and 2 good adjustment; TLI and CFI between 0.8–0.9 poor adjustment, and between 0.9–0.95 good fit; for the RMSEA between 0.05–0.10 acceptable and < 0.05 very good adjustment.

We adopted as the estimation method diagonally weighted least squares (DWLS) with polychoric matrices with Satorra-Bentler scaled chi square (S-B*χ*^2^) robust (Satorra & Bentler, 1994). This technique is appropriate for test models with ordinal categorical variables in small and medium size samples (*n* < 500) (Jöreskog & Sörbom, 1999). The internal consistency of the WHOQOL-OLD factors was checked by composite reliability (CR).

After AFCs were done, to estimate parameters of items of scale by item response package theory (IRT), ltm (Rizopoulos, 2006) and Mokken (Van der Ark, 2007) were adopted. The Mokken package showed monoticity of items and scale. This hypothesis considered the probability of one subject responding correctly to an item difficult to get right and having less difficulty or greater ease compared with a subject who had made an error on the difficult item. This propriety indicates that response to items is a theta function (Van der Ark, 2007), specifically for the WHOQOL-OLD instrument, which is not about getting an answer right or wrong but how satisfied with the expression of the item. The literature suggests that a Löevinger H (H for total scale and HS for each item) above 0.30 is a good indicator of monotonicity scale and items. In addition, the rho of de Mokken was used to evaluate whether data of the sample supported the IRT mathematical model. Values higher than 0.80 are indicators that data are reliable (Mokken & Lewis, 1982; Van der Ark, 2007).

Next, we compared the IRT model of constant discrimination (*a*_*c*_) [Rating Scale Model (Andrich, 1978)] with variable discrimination (av) [graded response model (GRM) (Samejima, 1969)]. For this, we used chi-square (*χ*^2^) to compare the likelihood rate of each model. In case of no statistically significant difference, for saving issues, we opted the simplest model, i.e., constant discrimination, in which all items have the same value for discrimination parameter (*a*) (Andrade, 2014). As a measure for adjustment of response standard of participants of GRM model, we investigated the use of the *χ*^2^ test, the differences between frequencies of standards of response observed, and predicted frequencies by the model. We adopted values lower than 3 as an indication of good adjustment because of the small difference between what was observed and predicted (Drasgow, Levine, Tsien, Williams, & Mead, 1995).

The hypothesis of local independence of the items was verified according to the recommendation of Jr Finch and French (2015), using the Yen’s Q3 statistic (Yen, 1993). Values greater than 0.2 suggest a violation of the local independence assumption.

Finally, the ltm package was used to estimate difficulty parameters [Response thresholds (j)] and discrimination (*a*) of items. Response thresholds (j) represents the behavior of items concerning probability of respondents choice of answers because of the theta level (in this study, theta represents QL score standardized in *z* score) and discrimination (*a*), the property that item has to achieve difference to respondents in function to theta level (Embretson & Reise, 2000). This means that individuals with different QL, in case of the item present good discrimination, they have a tendency to register different responses in this item. The IRT model adopted was the graded response model (GRM).

## Results

Table 1 presents adjustment indicators for two version of the WHOQOL-OLD scale. The first model is formed by the WHOQOL-OLD scale with Likert-type response (five response options, traditional version) and the second represents the WHOQOL-OLD scale with three options of response (reduced version). Both scales were tested for hexafactorial oblique structure, i.e., with six factors that are correlated among themselves.

**Table 1:** Adjustment indicators for two versions of the WHOQOL-OLD scale

Factorial models		*χ* ^*2*^ */df*	*TLI*	*CFI*	*RMSEA* *(CI 90%)*
*1*	5 types of response	692.49/237 = 2.92	0.87	0.89	0.07 (0.06–0.08)
*2*	3 types of response	371.825/237 = 1.56	0.94	0.95	0.04 (0.03–0.05)

Note: The models are not comparable

Considering that they are two independent samples, with different styles of answers, it was avoided to compare the adjustments of the two models. Therefore, the objective was to verify if the hypothesis of unidimensionality could be assumed. In this sense, it was verified that the adjustment indicators with three response options (second model) presented a good fit (*χ*^2^/df = 1.56, TLI = 0.94, CFI = 0.95, RMSEA = 0.04) and the model with five response options (first model) showed acceptable but poor fit (*χ*^2^/df = 2.92, TLI = 0.87, CFI = 0.89, RMSEA = 0.07) (Table 1) (Marôco, 2014).

**Fig. 1:**
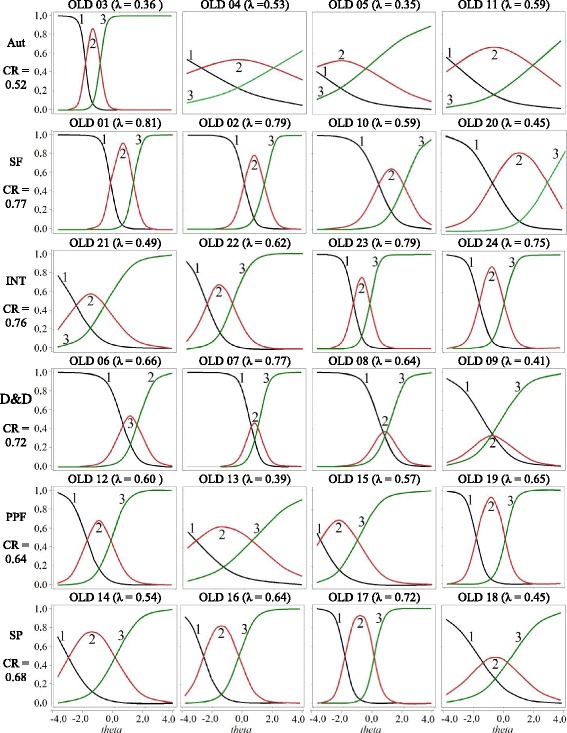
Description of the saturations, consistency, and WHOQOL-OLD Items Characteristic Curves (ICC)*.* The vertical axis represents the probability of choosing the answer for the item. “nothing” (01), “medium” (02), and “completely” (03) (Response options to items). λ – Lambdas. CR - composite reliability

**Fig. 2:**
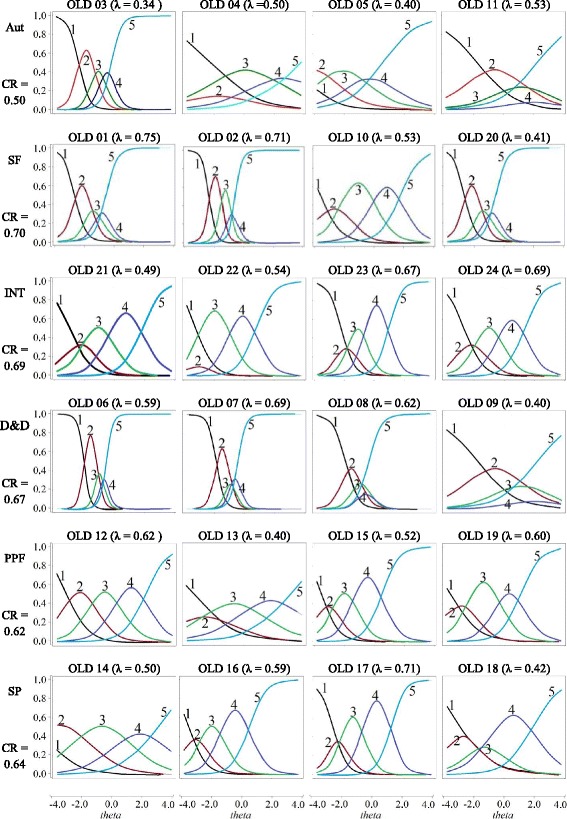
WHOQOL-OLD Items Characteristic Curve (original). The vertical axis represents the probability of choosing the answer for the item. “nothing” (1), “rarely” (2), “medium” (3), “very” (4), and “completely” (5). (Response options to items)

For the reduced version the composite reliability ranged from 0.52 (Aut factor) to 0.77 (FS factor) (Fig. 1). For the original version, it ranged from 0.50 (Aut Factor) to 0.70 (SF Factor) (Fig. 2).

We compare the behavior of the items in two response structures, in the six factors. The comparison of the variable discrimination models (GRM model) with constant discrimination showed a better fit to the model of Samejima (variable discrimination), with statistically significant differences for the six factors of the instrument in both versions (*p* < 0.05). In addition, the response patterns of the participants do not present problems that would be substantially relevant, i.e., *χ*^2^ greater than 3.0.

The results of the Mokken analysis for six factors corroborated the monoicity scale and items presenting Loevinger *H* equal to or greater than 0.3 and Rho > 0.80. One exception was the domain “Sensory function” (SF), with *H* = 0.28 (abbreviated version) and *H* = 0.27 (original version) and Rho = 0.72 (abbreviated version). Besides that, the statistic of Q3, to test the local independence of the items, in all combinations of items, for the two versions of the scale presented values superior to 0.2. The exception was for the autonomy factor, from the original scale, which presented a Q3 value of 0.22.

Table 2 shows parameters of WOQOL-OLD items with three response options. The factor that presented the lower mean and greater variance was “autonomy” (Aut) (*M* = 1.72, DP = 2.42), with one item that presented higher discrimination in the instrument [Item 3 (*a = 5.36*)] and, in the other items, the lowest discrimination. Mean of information of the “autonomy” factor was also the lowest of the six factors (*M* = 3.00; DP = 4.98). The factor that presented higher discrimination was “intimacy” (Int) (*M* = 2.33, SD = 1.04), and the one that presented the higher mean of information was “sensorial function” (SF). In relation to response hedge, four factors had first hedge mean (*j*_*1*_) lower than values of − 2 in the theta scale and no other factors overcame the mean of 1 in the second hedge.

**Table 2:** Description of the parameters of WHOQOL-OLD items (three answer options)

*Items*		Response thresholds		â	*Inf.*	*%*
		j_1_	j_2_			
*AUT*	OLD 03- Quanta liberdade você tem de tomar as suas próprias decisões	−1.98	− 1.00	5.36	10.48	100
	OLD 04- Até que ponto você sente que controla o seu futuro	−3.45	2.50	0.40	0.34	55.35
	OLD 05- O quanto você sente que as pessoas ao seu redor respeitam a sua liberdade	−4.43	− 0.14	0.54	0.54	61.63
	OLD 11- Até que ponto você consegue fazer as coisas que gostaria de fazer	−3.39	2.07	0.58	0.67	64.49
	*Média (DP)*	−3.31 (1.00)	0.85 (1.69)	1.72 (2.43)	3.00 (4.98)	70.00 (20.12)
*SF*	OLD 01- Até que ponto as perdas nos seus sentidos afetam a sua vida diária	−0.30	1.24	4.03	7.98	100
	OLD 02- Até que ponto a perda dos sentidos afeta a sua capacidade de participar em atividades	−0.04	1.32	3.07	5.75	99.98
	OLD 10- Até que ponto o funcionamento dos seus sentidos afeta a sua capacidade de interagir com outras pessoas	0.21	2.03	1.66	2.88	97.89
	OLD 20- Como você avaliaria o funcionamento dos seus sentidos	−2.84	0.79	−1.23	2.09	88.86
	*Média (DP)*	0.67 (1.46)	0.95 (1.21)	1.88 (2.29)	4.67 (2.70)	96.68 (5.30)
*INT*	*OLD 21-* Até que ponto você tem um sentimento de companheirismo em sua vida	−2.74	−0.52	1.18	1.80	88.9
	*OLD 22-* Até que ponto você sente amor em sua vida	−2.51	−0.73	1.82	3.18	96.54
	*OLD 23-* Até que ponto você tem oportunidades para amar	−1.39	−0.27	3.50	6.67	99.99
	*OLD 24-* Até que ponto você tem oportunidades para ser amado	−1.64	0.05	2.85	5.52	99.94
	*Média (*DP*)*	−2.07 (0.65)	−0.37 (0.33)	2.33 (1.04)	4.29 (2.20)	96.34 (5.21)
D&D	*OLD 06-* Quão preocupado você está com a maneira pela qual irá morrer	0.45	1.58	2.14	3.57	99.66
	*OLD 07-* O quanto você tem medo de não poder controlar a sua morte	0.34	0.98	3.10	4.91	99.99
	*OLD 08-* O quanto você tem medo de morrer	0.32	1.09	2.04	3.02	99.82
	*OLD 09-* O quanto você teme sofrer dor antes de morrer	−1.38	−0.16	1.10	1.50	95.55
	*Média (DP)*	−0.07 (0.88)	0.87 (0.73)	2.09 (0.82)	3.25 (1.41)	98.75 (2.14)
*PPF*	*OLD 12-* Até que ponto você está satisfeito com as suas oportunidades para continuar alcançando outras realizações na sua vida	−1.88	−0.24	1.96	3.50	99.13
	*OLD 13-* O quanto você sente que recebeu o reconhecimento que merece na sua vida	−3.45	0.66	0.70	0.88	72.27
	*OLD 15-* Quão satisfeito você está com aquilo que alcançou na sua vida	−3.65	−1.05	1.28	1.83	78.42
	*OLD 19-* Quão feliz você está com as coisas que você pode esperar daqui para frente	−1.85	0.11	3.06	6.03	99.93
	*Média (DP)*	−2.71 (0.98)	−0.13 (0.71)	1.75 (1.01)	3.01 (2.25)	87.43 (14.19)
*SP*	*OLD 14-* Até que ponto você sente que tem o suficiente para fazer em cada dia	−3.11	0.04	1.26	2.06	86.59
	*OLD 16-* Quão satisfeito você está com a maneira com a qual você usa o seu tempo	−2.59	−0.21	1.96	3.66	96.93
	*OLD 17-* Quão satisfeito você está com o seu nível de atividade	−1.92	0.03	3.39	6.72	99.96
	*OLD 18-* Quão satisfeito você está com as oportunidades que você tem para participar de atividades da comunidade	−1.66	0.52	0.97	1.45	92.17
	*Média (DP)*	−2.32 (0.66)	0.09 (0.31)	1.89 (1.08)	3.47 (2.35)	93.91 (5.84)

*Note: j*_*i*_ – Response thresholds; â – Discrimination; Inf. Information in the range of −4 to + 4 theta; % - percentage of total item information for the range of −4 to + 4 theta

Figure 1 presents items characteristics curves (ICC) of WHOQOL-OLD. The “autonomy” factor represents the item OLD 3 “How much freedom do you have in order to take your own decisions,” with great discrimination power but small theta interval. For example, each line of ICC represents the probability of support for one of the three possible responses because of the level of QL (theta). In the OLD 3 item, we observed that when the line that represents answer 1 (“nothing”) started to decline, changes in theta were quite sensible (representing discrimination ability) until they became most probable for the specific level (i.e., to *j*_*1*_ of item OLD 3 = − 1.98 theta) the alternative choice 2 (mean). The same occurs with transition between responses 2 and 3 (*j*_*2*_ of OLD 3 = − 1.00 theta). For OLD 3, approximately, after zero score of theta scale, a constant behavior is seen in line 3 (option of complete response), i.e., old people with thetas above of this point, even with different QL, support the same answer. Other items of the “Autonomy” factor (OLD 4, 5, 11) cover old people with greater QL, but with low discrimination.

Apparently, one specificity was in the D&D factor. The ICCs for this factor showed a polarization in behavior of answers: the answer “medium” had, in almost all thetas, less chance to be chosen than answers “nothing” and “completely” (Fig. 1).

Figure 2 presents the ICCs from the original WHOQOL-OLD version to investigate whether the behavior of the items in the factors would be different from the adapted version (Fig. 1). It is verified that in all factors, two or more items present response options with low probability of endorsement, regardless of theta. This would also indicate that a version with fewer options would be plausible. Regarding the autonomy factor item OLD 3 presented good discrimination, but the remaining items 4, 5, and 11 presented discrimination problems.

## Discussion

This study presented evidence of factorial validity in aversion of the WHOQOL-OLD scale using three response categories for older people from a countryside city in Paraíba, Brazil. Results found that use of this measure (three options for response) with hexafactorial oblique structure can be more acceptable than the original version. Another promising aspect of this version is that, unlike the studies by Leão (2012) and Chachamovich et al. (2008), which needed to change the number of items in order to find an acceptable adjustment, this study, without denying that it may be possible to find adaptations or insertions of content in future studies, did not need to remove any item from the original version of the scale. Thus, it represented only a change in the number of response options for each item. This can indicate that difficulty in finding stability in the factorial structure of WHOQOL-OLD lies not only in the problems of concept order that are linked to the complexity of the QL construct but also in the need to facilitate interpretation of options in answers from old people.

Approximately 80% of elderly participants in the two samples of this study had completed only elementary education. This aspect makes it difficult for them to understand scales with many answer options (Chachamovich et al., 2008; Fang et al., 2011; Fernandes & Santos, 2015; Matos, Mourão, & Coelho, 2016). Old people who lived in Northeast Brazil have cultural traits different from Brazilians of the South region, where the original version was evaluated and validated. One of these traits is level of education—old people from Porto Alegre (South region, Brazil) have, on average, a higher level of education than those from the Northeast region (Melo, Eulalio, Gouveia, & Silva, 2013; Melo, Ferreira, & Texeira, 2014).

After noting that reducing the answer options on this scale presented better adjustment, we investigated the behavior of these options regarding QL in these old people. The study showed that chance of discrimination of items in old people with different QL can be considered high because in case QL higher than 1.70 (Baker, 2001), this number indicates high quality discrimation in terms of quality of life. However, according to the author (Baker & Kim, 2017), these indicators were proposed for dichotomous items, and cutoff points considered for these parameters need to increase because of the number of options on the studied scale. There is a need to consider the tendency of increased discrimination of items seen with a higher number of possible answers because, naturally, these respondents would have more chances to be divided according to answer options (Cavalcanti, Melo, Medeiros, Oliveira, & Gouveia, 2016; Tezza, Bornia, & Andrade, 2011). For this reason, comparison of discrimination of scales is sensible because they contain items with a number of different answer options.

Although the categories “autonomy” (Aut) and “sensorial function” (SF) did not differ from other facets, they were the ones that presented smaller means of discrimination. Therefore, this property represents the ability of an item to differentiate among respondents because of changes in theta (in this study, represented by QL) (Samejima, 1969), and because of these changes, the higher the discrimination, the greater the sensibility of the item for placing subjects in its real position on the QL scale. There are at least two problems that can be emphasized for factors with low discrimination. The first would be interference in studies in which the use of the score for the factor for covariance studies, because subjects with different QL can be classified in the same score, would increase residues of the model, and generate correlation with magnitudes that reflect less loyal relationship between variables. In this case, the construct would not vary when and where it should vary. In addition, the less variability there is, the more difficult the investigation becomes and the more difficult the implementation of public policies that need to absorb and address different demands of subjects with different QL. Therefore, it would be difficult to differentiate elderly persons in terms of QL.

Other important information that IRT offers is an indication that items might not provide the same information condition to elderly persons that QL is different. In this regard, a warning would be made about the observation of the mean of answers hedges, in which we observed hedges (mainly the *j*_1_) that cover most of the low portion of theta. This indicates that the scale would evaluate fewer mistakes from elderly persons with worse QL. This is more evident for the factor “Social and intimacy participation,” in which hedge “two” (*j*_2_) presents lower means of scale.

We also observed that the “intimacy” factor had a higher mean of discrimination because in addition to offering more safety information for elderly persons with low QL, it could precisely discriminate and quantitatively provide more information on the construct. In this regard, in the study of Leão (2012), which used focal groups of old people to investigate factors and items of WHOQOL-OLD, the intimacy with family had an emphasis in speech of elderly person, who attributed the opportunity of loving and being loved as critical for their health and improved their QL. The importance of love was also found in other studies (Fernandes & Andrade, 2016; Teixeira, 2013). These elements are present in factor items such as “Intimacy” (Ex. item 23 – “To what extent do you have the opportunity of loving” (more discrimination of the factor). However, lack of valorization, lack of attention, and abandonment would be among the largest fears and risks for QL in the study of Leão (2012). Perhaps given the importance placed on this question in assessment of life aspects by elderly persons, the item would be better able to distinguish between different QLs in the “intimacy” factor. If loving or being loved is experienced with greater need and priority, small changes in these aspects may be felt with more intensity and satisfaction, therefore justifying the higher mean of discrimination of this factor.

Table 2 and Fig. 1 show that the “autonomy” factor presents (except item 3) items with discrimination problems and information. For this reason, specifically for this factor, we verified that ICCs of the scale with five options of answer (Fig. 2). This comparison can indicate whether the problem can reduce the number of answer options from five to three. Results suggest that this hypothesis may be excluded because the same items in Fig. 2 present discrimination problems and little information. This suggests that the problem may be more related to the content of the items.

In this sense, items with other content regarding autonomy would be tested. Leão (2012) found the financial component was a strong indicator of old people’s autonomy. In studied discourses of old people by Leão (2012), for example, “We need to have a certain financial dependency because without financial independency, we are nothing.” The WHOQOL-OLD instrument has the aim to evaluate QL in old people from different contexts. This requires the need to develop items, at times generic content, to cover the greatest number of population members, such as in item 11 (“To what extent you can do things you would like to do”). When someone does what he/she likes, that status can depend of other previous conditions, such as health and finances; therefore, it would be possible to test this factor with items that would place these conditions in a form more directly related to autonomy (for example, the hypothetical item “My economic condition allows me to do what I like to”).

The factor D&D also presented specificity. The second option for answer items (red curve of Fig. 2) does not give information on quality of life in D&D. Curves of items 6 and 7 show that a small parcel of theta has more changes in choosing the option of the answer “medium” (red curve). For items 8 to 9, the greater change does not exist. For example, for item 9, an elderly person with specific QL in D&D can have more changes in choosing option 0 (“nothing”), and another elderly person, with QL close to the anterior, would have more changes in choosing response 2 (“completely”). This shows that elderly persons tend to be not at all satisfied or “completely” satisfied in relation to D&D. Other authors also verify this polarization for this factor (Melo et al., 2013).

According to study of Leão (2012), elderly persons from Northeast Brazil report that they do not fear death or do not think too much about dying. These finding agree with items of the D&D factor that are related to the manner of death, fear over having no control over how one dies, or even feeling pain before dying. Still, regarding this respect, Menezes e Lopes (2014) found ambiguous reports on fear of death, showing that old people, who are closer to death, bring a dynamic of feeling that, within the same person, oscillate constantly between fear and resignation. This variation of feeling could be explained by ambiguity of reports on death, found by authors, and polarity of satisfaction in the D&D factor in the WHOQOL-OLD instrument.

The majority properties that instrument presented were positive. For this reason, we understand that the version of the scale that uses three answer options shows evidence that the instrument can be applied in the elderly population from Northeast Brazil and even to the overall Brazilian elderly population. Therefore, same results show that this does not mean that the scale needs to be adjusted in order to improve it, mainly because, conceptually, QL is multidetermined and subjective and it is difficult to avoid the variability that exists in each cultural context (Gordia et al., 2011; Vilar, 2015).

In our study, an aspect of moderation that needed to be taken into account is the non-probabilistic sample used. This limitation increases the representative error and reduces trustworthiness when compared to factorial models of two versions of the WHOQOL-OLD scale. Further studies need to be done with the reduced version of the WHOQOL-OLD. There is a need to check whether different education levels affects the consistency of elderly persons’ responses. This can be done by different functional techniques.

## Conclusions

Finally, we suggest adoption of number 0 to represent the alternative of the answer “nothing” of 1 to answer “medium” and 2 to answer “completely.” This makes it easy to calculate the standardized scores (range, 0 to 100) proposed by the original version of WHOQOL-OLD, based on the classic theory of tests. Posteriorly, to multiply answers by 50 would be enough to avoid more calculations. After multiply and elaborate factors, because of items observed in Table 2, the score would be used to compare QL in studies that use both the original scale and the reduced scale because metrics were standardized from 0 (for dissatisfaction with QL) to 100 (complete satisfaction with QL).

Further studies should be performed both to facilitate understanding of QL among elderly persons and to improve measurement of QL. This study may contribute to development of other studies in this area of knowledge.
